# From microbes to molecules: unveiling host-microbe interactions with spatial metabolomics

**DOI:** 10.1038/s44320-025-00129-x

**Published:** 2025-06-24

**Authors:** Malin Stüwe, Lars-Erik Petersen, Manuel Liebeke

**Affiliations:** 1 Department for Metabolomics, Institute for Human Nutrition and Food Science, Christian-Albrecht-University Kiel, Kiel, 24118 Germany; 2 Max Planck Institute for Marine Microbiology, Bremen, 28359 Germany

**Keywords:** Evolution & Ecology, Metabolism, Microbiology, Virology & Host Pathogen Interaction

## Abstract

This Comment discusses key aspects in microbial spatial metabolomics and calls for a deeper exploration of microbial systems to advance chemical ecological concepts, host-microbe interactions, and metabolic mechanisms in microbial communities.

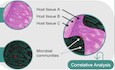

## Teaser

Spatial metabolomics is transforming our understanding of microbial systems by uncovering the localized molecular dynamics within complex microbial communities. By using advanced mass spectrometry imaging techniques, researchers can now visualize metabolites at the micron scale and show how microbes interact, form biofilms, and influence their environments at unprecedented levels of detail. These approaches not only provide molecular insights they also highlight the need of integrating activity measurements and temporal analyses to capture the dynamic nature of microbial assemblages. Incorporating these dimensions in future will enable a deeper understanding of microbial interactions over time, bridging the gap between static snapshots and the evolving processes that drive microbial ecosystems. This commentary explores important aspects in microbial spatial metabolomics, underscores the need for activity and time-resolved studies, and advocates for a deeper exploration of microbial systems to advance chemical ecological concepts, host–microbe interactions, and metabolic mechanisms in microbial communities.

## Why metabolites and why location matters

**Figure 1:**
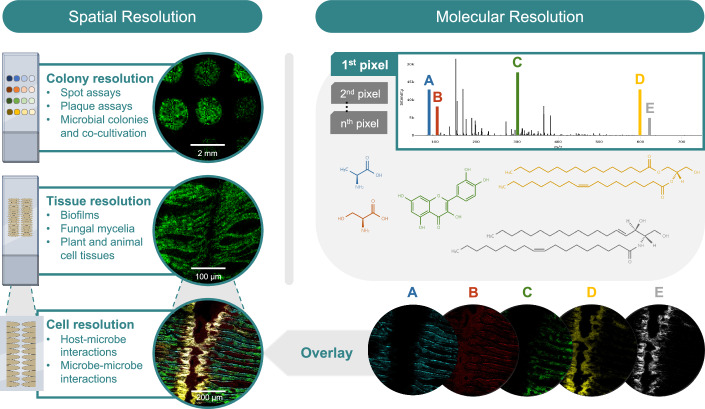
Resolution matters for microbial spatial metabolomics. Spatial and molecular resolution are important measures to enable spatial metabolomics for microbial systems. Spatial resolution describes the smallest area on a sample that can be individually analyzed and resolved to produce a distinct mass spectrum. Colony, tissue and cell resolution show examples of a spot assay at 100 µm, tissue at 10 µm and tissue at 2 µm pixel size, respectively. Molecular resolution in MSI mainly depends on mass accuracy to differentiate between molecules (e.g., amino acids, lipids, small metabolites).

Activity and location is key. Metabolites, the common language between cells, are involved in communication, defense and nutritional exchange in microbe–microbe and host–microbe interactions. The spatial distribution of metabolites in microbial systems is influenced by active cells and depends on the interactions these cells perform and consequently needs to be mapped in relation to each individual cell type. However, a reliable approach to probe the complex metabolic and site-specific phenotypes of microbes requires pinpointing individual metabolites on the micron scale, a key challenge in microbial chemical ecology. Spatial metabolomics using mass spectrometry (MS) advanced tremendously during the last years and offers a window into metabolic interactions of microbial systems ranging from biofilms to host–microbe assemblages. This in situ method enables real observation of phenotypes on the metabolite level. The primary technological driver has been spatial resolution (the pixel size, defined by the ability to distinguish two discrete measurement points), followed by molecular resolution (the ability to differentiate metabolites based on structural features and exact mass) (Fig. 1). While current mass spectrometry imaging (MSI) techniques can achieve spatial resolutions between 10 and 5 µm, only prototype systems can reach below and achieve 1 µm pixel sizes (Potthoff et al, 2024). The most commonly used spatial metabolomics methods are matrix-assisted laser desorption ionization (MALDI) and desorption electrospray ionisation (DESI), which offer high molecular resolution by measuring intact metabolites. In contrast, methods like secondary ion MS provide outstanding spatial resolution but less molecular information (Petras et al, 2017). As the field transitions to higher cellular resolution, there is a growing need to visualize not only structural features but also the full range of chemicals and metabolites. Currently, this combination is best achieved with MALDI-MSI in spatial metabolomics.

This raises a fundamental question: Which metabolites can we measure and map at micron resolution using MALDI-MSI? A detailed multi-lab MSI investigation demonstrated the broad coverage of core bacterial metabolism (*E. coli*), highlighting the capabilities of spatial metabolomics to capture a wide array of metabolic pathways (Saharuka et al, 2024). Here, MALDI-MSI excels at detecting various classes of metabolites, including lipids, small peptides, amino acids, organic acids, nucleotides, and secondary metabolites. Lipids are among the most readily detectable molecules due to their cellular abundance and high ionization efficiency, making it possible to map the distribution of phospholipids, glycerolipids, and sphingolipids with high spatial precision. In addition, small peptides and amino acids, key players in primary metabolism, can be identified and spatially resolved, offering insights into cellular functions and metabolic interactions. Although carbohydrates and other small metabolites are challenging to detect with MALDI-MSI due to their poor ionization efficiency, advances in matrix chemistry, sample preparation and the use of post-ionization are improving their detection. Natural products, which play crucial roles in microbial communication and defense, can also be visualized, providing valuable insights into ecological interactions and microbial behavior. This highly diverse detection capability positions MALDI-MSI as a powerful tool for exploring the spatial organization of microbial metabolism and understanding the intricate chemical landscape of microbial communities.

Microbes in high abundance form structured communities, such as biofilms or microbial consortia, where metabolic interactions occur at the micron scale. Capturing these interactions requires imaging techniques with pixel sizes that match the scale of individual cells. For example, recent studies have mapped the distribution of lipids, peptides, and secondary metabolites in bacterial colonies, uncovering spatial gradients that correlate with metabolic activity and chemical ecological function (Shen et al, 2024).

However, the resolution of these insights is directly tied to the pixel size used for MS imaging. Larger pixel sizes may obscure critical details of microbial heterogeneity, while higher-resolution imaging (below 1 µm) can reveal previously hidden patterns of metabolite distribution. Figure 1 illustrates a comparison of spatial metabolomics data at different pixel sizes and emphasizes how higher resolution can resolve finer details of microbial metabolic landscapes. This underscores the need for continued advancements in spatial resolution to fully exploit the potential of spatial metabolomics in microbial research. By achieving higher resolution, we can better observe the intricate chemical interactions that drive microbial behavior, cooperation, and competition.

## Microbial secrets can be revealed with spatial metabolomics

Spatial metabolomics offers a powerful lens for uncovering the hidden chemistries within microbial communities. By preserving the spatial organization of metabolic processes, this approach provides direct insight into microbial interactions within complex ecosystems. Rather than inferring function from homogenized samples, spatial metabolomics captures the metabolic phenotypes occurring directly in microbial colonies or environmental samples.

One of the key insights comes from examining metabolite-driven microbial interactions, such as competition and cooperation. Spatially resolved data reveal how microbes localize the production of antibiotics, siderophores, or signaling molecules to strategic zones, often at interfaces between colonies, highlighting molecular strategies for niche defense or signaling (Schleyer et al, 2019; Watrous et al, 2013). These localized interactions shape microbial community structure, succession, and resilience in response to environmental stressors.

Another critical area is biofilm formation, where spatial metabolomics can resolve the chemical gradients that define biofilm architecture and function. Within these densely packed microbial communities, metabolites such as quorum-sensing signals, extracellular matrix components, or metabolic waste products are heterogeneously distributed. Spatial analysis has shown that metabolic zoning within biofilms supports specialized roles like nutrient cycling or defense (McCaughey et al, 2024).

Importantly, spatial metabolomics not only identifies which metabolites are present, but where and when they are active. This enables a more nuanced view of microbial life that accounts for spatial complexity, environmental feedback, and dynamic interactions like in colonies or biofilms. Crucially, spatial metabolomics reveals that metabolite function is highly context-dependent, shaped not just by molecular composition but by the specific spatial and temporal environments in which microbes exist.

## Biological insights into host–microbe interactions provided by MSI

Most of our current understanding of microbial metabolism comes from studies on isolated species or simplified co-cultures that characterized the metabolome and lipidome of individual or interacting microbes in controlled environments. While these approaches have revealed important metabolic capabilities and interspecies interactions, they fall short of capturing the full complexity of microbial life within host tissues. Mammalian symbiotic tissues can host up to hundreds of bacterial species which are spatially structured and influenced by host-derived factors. To fully understand microbial function and host–microbe interactions, it is essential to investigate these communities within native tissues where ecological complexity and host influences are preserved.

A powerful approach to resolve host–microbe associations in their native tissue environment is the combination of MSI with 16S rRNA fluorescence in situ hybridization (FISH). FISH uses fluorescently labeled, taxon-specific oligonucleotide probes to identify and localize bacterial cells within tissue sections. Probes can be designed based on the study objectives and can range in specificity from phylum to species level. The combination of 16S rRNA FISH and MALDI-MSI on the same tissue section allows to directly link microbial identity to metabolic activity (Bourceau et al, 2023). Notably, MSI can reveal microbial lipid biomarkers, facilitating the development of reference libraries that may allow taxonomic inference from metabolite profiles alone, bypassing the need for separate nucleic acid-based identification in the future (Chen et al, 2025). Although comprehensive biomarker databases have been developed from cultured strains, it is less known how well these profiles represent microbial metabolism in situ, especially for unculturable taxa and in host-associated environments. Because many microbes cannot be grown in the lab, their metabolic activities remain poorly understood. Spatial metabolomics overcomes this limitation by enabling direct detection of microbes within tissue and revealing the molecules they produce in their natural context.

**Figure 2:**
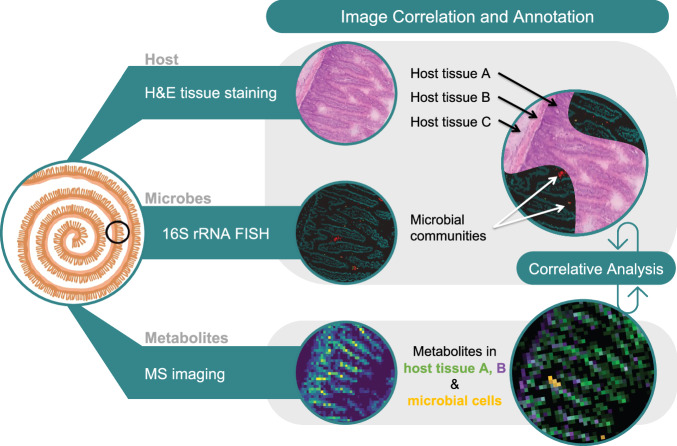
A combination of different imaging methods is needed to fully map the chemical landscape of host–microbe interactions in the tissue context. This figure illustrates a multimodal imaging approach combining H&E tissue staining to identify host cell morphology, 16S rRNA FISH to localize microbial populations and MALDI-MSI to map metabolites involved in host–microbe interactions. The workflow can be applied to any tissue and is illustrated for the murine small intestine.

Alterations in microbiome composition are frequently associated with host health outcomes, particularly in relation to metabolism, immunity, and disease susceptibility. Microbial metabolites such as short-chain fatty acids, amino acid catabolites or secondary metabolites are important players in the crosstalk of host and associated microbes. MSI enables the in situ detection and spatial mapping of these compounds within tissues, offering direct insight into how microbial activity shapes the local biochemical landscape. In addition to MSI and FISH, H&E staining can be performed to assess potential changes in host histological phenotypes that are linked to the presence of specific bacteria or bacterial metabolites (Fig. 2). In the future, this will improve our understanding of how bacterial communities shape local metabolic microenvironments and influence host health and tissue phenotype.

Despite its promise, studying metabolic interactions within complex, multi-species communities in vivo remains a major challenge due to both biological complexity and technical limitations. The high diversity of microbes in native tissues along with resolution and sensitivity constraints of current MSI technologies make it difficult to resolve metabolite distributions at the level of individual microbial populations. Overcoming these barriers will be essential for understanding how microbial communities contribute to host physiology and disease outcomes.

## Why is spatial metabolomics not yet at the forefront of methods for microbial systems?

Many worlds unite when spatial metabolomics is applied to microbial systems: microbiology, chemistry, ecology and bioinformatics. It is apparent that these diverse science fields use different languages and concepts which results in communication barriers. Such a situation calls for common goals, understood by each discipline. For spatial metabolomics, this means uniting chemical and biological approaches to characterize the diversity and function of host-associated microbiomes. Complex chemical information needs to be presented to biologists in an easy-to-approach way. Finding standardized protocols can be challenging since the diverse setups for MSI experiments along with the variety of chemical matrices available make sample processing highly individual. Nevertheless, openly sharing these workflows among the MSI community will make them approachable for new people in the field as well.

The broad implementation of MSI in microbial systems is furthermore hindered by technical limitations. One major challenge is the low concentration of metabolites within tissues and microbial cells, which continues to test the sensitivity limits of modern mass spectrometers. This limitation also complicates in situ MS2-based fragmentation, a crucial step for accurate metabolite identification. In addition, many of the detected molecular features are not annotated as known metabolites and therefore cannot be put into ecological context, as we do not know their identity. This situation is further complicated by the diverse metabolism of 1000s of microbial species. Even in the case of annotated features, many metabolite maps are less meaningful if they miss cell type identification using correlative imaging. Commercial MALDI-MSI systems do not operate on single-cell level (<2 µm) but instruments continue to develop fast and might overcome current spatial and molecular resolution barriers. As of today, custom-built MALDI-MSI systems are able to retrieve metabolite signals from very small pixel sizes (1 × 1 µm) (Potthoff et al, 2024).

## The future of spatial metabolomics for microbial systems

Being able to observe snapshots of phenotypic behavior and map chemical interactions between cells is a major advancement, still MSI lacks an important biological factor: time. The temporal resolution is a future wish and can so far not be covered by spatial metabolomics approaches as the majority of methods are destructive or too harsh for cells to grow further after a metabolic snapshot was taken. An exception are experimental setups that are capable of recording the evolving metabolic footprint of microbial interactions over time. Plaque assays, for example, unfold the metabolic landscape generated during virus infection of host cells in high spatiotemporal resolution, thereby documenting this continuous process not unlike growth rings of trees (Schleyer et al, 2019).

A big advantage of analyzing metabolites through MS lies in the ability to detect the incorporation of heavy isotopes into molecules. This offers a powerful and sensitive means to trace metabolic activity within cells and microbial communities. By introducing isotopically labeled substrates such as D_2_O, ¹³C-glucose, or ^15^N-amino acids, scientists can follow the metabolic fate of these compounds, effectively providing a snapshot of which pathways are active and which organisms are metabolically engaged. Importantly, the spatial distribution of metabolic activity can be visualized by MSI, providing high-resolution maps of biochemical processes in situ (Schwaiger-Haber et al, 2023; Wang et al, 2022). This approach can also reveal the presence of metabolites with very high flux and low in situ concentrations as the isotope label as an imprint of the reaction. Using stable isotopes not only reveals the dynamics of nutrient fluxes but also allows for distinguishing between dormant and active cells in the three-dimensional space. Overall, the integration of isotope labeling with MS-based metabolomics and imaging creates a versatile toolkit for probing functional activity in microbiomes and tissues.

Much progress has been made for both spatial transcriptomics and proteomics, although the microbial component is often not covered. If integrated with these two techniques, however, spatial metabolomics will contribute to a multi-omic view on microbial interactions, linking spatially resolved chemistry to proteins and active genes. We see a new era for spatial multi-omics microbiology and at its forefront will be metabolomics with the ability to listen into the chemical language of cells with their microbial community partners or surrounding host cells and tissues.

Spatial metabolomics is rapidly advancing, with efforts focused on improving its sensitivity, resolution and throughput. As instrumentation advances and spatial resolution improves, MSI for microbiology is on its way to offer even deeper insights into the functional and dynamic nature of host–microbe interactions. Its capability uniquely bridges microbiology and host biology, allowing for in situ analysis of metabolic processes in complex cellular environments. Integration with other spatial omics and imaging technologies is key to giving context to the findings from multipartite assemblages. Together, these tools will enhance our understanding of microbial metabolism in different habitats, ranging from biofilms to intracellular pathogens. The advances in spatial metabolomics will enable a deeper understanding of microbiome research and promise to reveal novel mechanisms in the interactions between microbes and other organisms. As such, spatial metabolomics is essential for decoding the chemical language that underpins microbial ecosystems from environmental microbiomes to infection sites.
